# The effect of anode potential on current production from complex substrates in bioelectrochemical systems: a case study with glucose

**DOI:** 10.1007/s00253-020-10547-6

**Published:** 2020-04-04

**Authors:** Fei Zhao, Elizabeth S. Heidrich, Thomas P. Curtis, Jan Dolfing

**Affiliations:** School of Engineering, Newcastle University, Newcastle-upon-Tyne, England NE1 7RU UK

**Keywords:** Bioelectrochemical system, Anode potential, Glucose, Current production

## Abstract

Anode potential can affect the degradation pathway of complex substrates in bioelectrochemical systems (BESs), thereby influencing current production and coulombic efficiency. However, the intricacies behind this interplay are poorly understood. This study used glucose as a model substrate to comprehensively investigate the effect of different anode potentials (− 150 mV, 0 mV and + 200 mV) on the relationship between current production, the electrogenic pathway and the abundance of the electrogenic microorganisms involved in batch mode fed BESs. Current production in glucose-acclimatized reactors was a function of the abundance of *Geobacteraceae* and of the availability of acetate and formate produced by glucose degradation. Current production was increased by high anode potentials during acclimation (0 mV and + 200 mV), likely due to more *Geobacteraceae* developing. However, this effect was much weaker than a stimulus from an artificial high acetate supply: acetate was the rate-limiting intermediate in these systems. The supply of acetate could not be influenced by anode potential; altering the flow regime, batch time and management of the upstream fermentation processes may be a greater engineering tool in BES. However, these findings suggest that if high current production is the focus, it will be extremely difficult to achieve success with complex waste streams such as domestic wastewater.

**Electronic supplementary material:**

The online version of this article (10.1007/s00253-020-10547-6) contains supplementary material, which is available to authorized users.

## Introduction

Bioelectrochemical systems (BESs) have been proposed as an energy-neutral wastewater treatment technology, but commercially acceptable performance of BESs treating wastewater has yet to be realized (Dhar and Lee 2014; Li et al. 2013). One of the stumbling blocks is the poor current production, and therefore energy recovery, from the complex substrates available in waste. With acetate, a direct substrate for the electrogenic bacteria that drive BESs, high current production has been achieved (e.g. ~ 0.25 mA/cm^2^) (Kiely et al. 2011). However, with complex substrates, this direct electrogenic path is generally not available (Freguia et al. 2008). The complex substrate must be broken down in various stages to different components, which involves a network of microorganisms. The current production with these substrates is erratic and usually low (e.g. ~ 0.017 mA/cm^2^ with starch) (Velasquez-Orta et al. 2011). More information on the complex degradation pathway and intricacies of, and between, the organisms in BES processes is urgently needed to drive the field forward.

One of the parameters within BESs that we as engineers can influence, which may stimulate the rate of current production, is the anode potential. In theory, electrogenic microorganisms can obtain more energy if the anode potential is high (Wagner et al. 2010). Thus, a high anode potential should stimulate the degradation of complex wastes. This could be by directly promoting the growth of the electrogenic microorganisms via high energy supply (Wei et al. 2010). Ishii et al. (2014) have shown that the substrate degradation profile in BESs was affected by the anode potential, indicating that anode potential generates a stimulus that propagates throughout the network to the organisms that function upstream from the electrogenic microorganism An improvement in the degradation of a complex waste would result in the increase of (i) the current, that is, the rate at which electrons are produced; and (ii) the coulombic efficiency, that is the efficiency with which the electrons in the substrate are converted into current. The latter may be of particular importance as with complex wastes where part of the substrate may be converted into dead-end products that never reach the electrogenic stage. Several studies have shown that anode potential affects the coulombic efficiency in BESs, demonstrating the variation of final products and electron sinks of substrate conversion at different anode potentials (Freguia et al. 2008; Hari et al. 2016).

The effects of anode potential on current in BESs have also been reported and various electrogenic microorganisms that related to high current production have been identified. (Dennis et al. 2016; Ishii et al. 2014). These results are topical, but more information is needed to quantitively describe the effect of anode potential on current in BESs. A critical question is “what is the key stimulus that determines the performance of electrogenic microorganisms at different anode potentials?” A second critical question relates to the maximum current that can be achieved with complex substrates that are not used directly by the electrogens, but need to be converted first by non-electrogens to intermediates that can subsequently be used by electrogens. Following Monod kinetics, electrogens only produce maximum current (*V*_max_) when concentrations are sufficiently high (Rittmann and McCarty 2012). Thus, the question is to what extent do the concentrations of those intermediates allow *V*_max_, or phrased differently, do the upstream microorganisms produce electrogenic intermediates at sufficient rates to allow electrogens to function at maximum capacity?

To answer this question, the upstream production and downstream consumption routes and rates for electrogenic molecules need to be identified. Glucose can be used as a model substrate as it exhibits complex degradation pathways involving multiple intermediates and therefore electrogenic constraints. Furthermore, contrary to complexity of, and in, wastewater, glucose degradation intermediates and products are well characterized and easy to measure (Lee et al. 2008; Lee and Rittmann 2009). Typical glucose degradation intermediates and products such as volatile fatty acids are often abundant in glucose-fed system, while other important intermediates such as pyruvate and hydrogen do not often accumulate to measurable levels (Lee et al. 2008; Stams and Plugge 2009). It is necessary to investigate the fate of all the major glucose degradation intermediates and products to attain a full picture of electrogenic pathway in glucose-acclimatized BESs. Additionally, the electrogenic role and degradation products of glucose degradation intermediates and products as well as pyruvate and hydrogen need to be individually evaluated.

In this study, we evaluate the effect of anode potential on current production in BES and connect this to the pathway of glucose degradation. To achieve this, we acclimatized a series BESs to glucose at three different anode potentials (+ 200 mV, 0 mV and − 150 mV vs standard hydrogen electrode (SHE)). Then, we systematically (1) identified the major electrogenic molecules and evaluated their minimum concentration needed for saturated current production; (2) identified the pathway of the degradation of glucose and its intermediates and evaluated the effect of the rate of these pathways at different anode potentials; (3) investigated the effect of anode potentials on the relationship between the abundance of known electrogenic microorganisms and current production.

## Materials and methods

### Reactor setup

Two-chamber H-type BESs reactors were constructed by combining two 300-mL borosilicate glass bottles with 250-mL individual working volume (Duran, Germany) as anodic and cathodic chambers (Fig. S8). These were separated by a Nafion 117 membrane (Sigma-Aldrich, USA). Graphite plates sized 5 cm × 2.5 cm × 1 cm (Olmec, UK) were used as anode; these were soaked in 10% HCl overnight and rinsed with deionized water to remove the residuals on the surface. Platinum meshes sized 5 cm × 2.5 cm (Ti-shop, UK) were used as cathode after rinsing with deionized water. The electrodes were connected with a 1.0-mm-diameter stainless steel wire (Goodfellow, UK). The ohmic resistance of each electrode setup was tested with a voltammeter to ensure that the resistance was less than five ohms.

### Media and buffer

Settled sewage from the Tudhoe Mill domestic wastewater treatment plant (Durham, UK) was used as inoculum in the anodic chamber. Sewage inocula were used in a ratio of 1:5 with anodic media for the initial acclimation. The anodic media contained disodium phosphate (Na_2_HPO_4_, 4.58 g/L), sodium dihydrogen phosphate (NaH_2_PO_4_·2H_2_O, 2.77 g/L), ammonium chloride NH_4_Cl (1 g/L), trace element solution (10 mL/L) and vitamin solution (10 mL/L) (Gimkiewicz and Harnisch 2013). Glucose (0.6 g/L) was used as a substrate for anodic biofilm acclimatization in experimental reactors and acetate (0.82 g/L) in control reactors. Cathodic buffer contained disodium phosphate (Na_2_HPO_4_, 4.58 g/L) and sodium dihydrogen phosphate (NaH_2_PO_4_·2H_2_O, 2.77 g/L). The final pH in anodic media and cathodic buffer was 7.0. Both solutions were sparged with N_2_ (99%) for 30 min before use.

### Reactor operation

Experimental reactors and controls were acclimatized at three different anode potentials: − 150 mV, 0 mV and + 20 0 mV vs SHE. Reactors with each anode potential were set up and run in duplicate, six reactors were used in total. Quad potentiostats (Whistonbrook, UK) were used to control the anode potential of these reactors. AgCl/Cl electrodes (+ 197 mV vs SHE) were used as a reference electrode (MF-2052, BASi, USA). The distance between the reference electrode and anode was kept to ~ 10 mm in order to minimize potential loss. Current production was recorded once per minute by version 3 data logging software (Whistonbrook, UK). Polarization curves were obtained by measuring the peak current until stable production in eight individual cycles at anode potentials from − 300 to + 600 mV.

### The individual intermediates and product tests in glucose-acclimatized BESs

To identify the major electrogenic molecules and the degradation pathway of glucose in glucose-acclimatized BESs, each intermediate molecule was fed individually into the reactors and the current and substrate removal were measured. The initial concentration of organic substrates for each test was 20 mol of carbon/L, which was equivalent to 0.82 g/L sodium acetate, 1.36 g/L sodium formate, 583 mL/L ethanol, 0.64 g/L sodium propionate and 0.73 g/L sodium pyruvate. Tests with H_2_ and CO_2_ (80:20) were also performed. All tests were performed at both acclimation anode potential and open circuit potential for 9 h except the hydrogen tests, which were run for 2 h. After each test, the reactors were operated with 0.6 g/L glucose overnight at their acclimatized anode potential to stabilize the anodic microbial communities.

### The evaluation of minimum availability of acetate and formate to reach maximum current production in glucose-acclimatized BESs

To estimate the minimum concentrations of acetate and formate needed for maximum current production in glucose-acclimatized BESs, peak currents were measured at a range of different concentrations of sodium acetate and sodium formate and concentrations used in individual cycles ranged between 0.2 and 1.8 g/L of sodium acetate and between 0.3 and 5.4 g/L of sodium formate. The peak current production at each concentration was associated with the corresponding concentration by nonlinear regression and used to obtain *K*_*s*_ values based on the Monod kinetics:

1 $$ \frac{1}{V}=\frac{K_s+\left[S\right]}{V_{\mathrm{max}}\ \left[S\right]} $$

where *V* is the current production (mA/cm^2^), [*S*] is the concentration of acetate or formate (mM) and *V*_max_ is the saturated current production (mA/cm^2^). *K*_*s*_ is the substrate concentration when the current production is half of *V*_max_ (mM) (Rittmann and McCarty 2012).

### Chemical analysis

The chemical composition of the liquid samples of influent and effluent in the tests mentioned above was analysed. TOC (total organic carbon) was determined with a total carbon analyser equipped with an ASI-5000A autosampler (TOC-5050A, Shimadzu, Japan). COD (Chemical Oxygen Demand) of each sample was measured with COD kits (25~ 1500 mg/L, Merck & Co. Inc., USA) according to the manufacturer’s protocol. Volatile fatty acids (VFAs) were determined by an ion exchange method (Manning and Bewsher 1997). Samples were mixed with oxysulfonic acid (1:1; v:v) and sonicated for 40 min to remove dissolved carbonate. Then, the treated samples were measured on an ion chromatograph equipped a 4 × 250 mm Ionpac ICE-AS1 column, with 1.0 mM heptafluorobutyric acid as eluent (IC, Dionex ICS-1000, Thermofisher, USA). The injection was conducted by a loop with a volume of 10 mL and a flow rate of 0.16 mL/min. Cation regenerant solution was 5 mM tetrabutylammonium hydroxide in the Dionex Anion Micro Membrane Suppressor (AMMS-ICE II). Glucose, pyruvate, ethanol and lactate were quantified with assay kits (Megazyme, Ireland) and a microplate reader at 340 nm (SpectraMax M3, USA) according to the manufacturer’s protocol.

Gas samples in the headspace of the reactors were taken with 100-μL gas-tight syringes (SGE Analytical Science, Australia). Hydrogen was determined with a Trace Ultra GC (Thermo Scientific, USA) equipped with a Restek Micropacked 2 m Shin carbon column with argon as the carrier gas. The detection procedure was to heat up the oven to 40 °C followed with ramping from 110 °C with an interval of 30 °C per min until 200 °C for 1.5 min. Methane (CH_4_) in the headspace of the reactors was determined by a Carlo Erba HRGC S160 GC equipped with an FID detector and a 0.32-mm-diameter HP-PLOTQ column with a length of 30 m. The temperature of the oven was set at 35 °C.

### Microbial community analysis

The microorganisms on the anodes were sampled and extracted with a sterilized stainless steel scratcher and preserved in 50% ethanol at − 20°C. The DNA of the anodic microbial communities was extracted by the QBiogene FastDNA spin soil kit (MP Biomedicals, UK).

To sequence the microbial communities, the V4 and V5 regions of 16S rRNA gene of the extracted DNA samples were amplified by polymerase chain reaction (PCR), using the forward primer 515f: 5′-*GTGNCAGCMG CCGCGGTAA*-3′ and the reverse primer 926r: 5′-*CCGYCAATTYMTTTRAGTTT*-3′ using the Roche Fast Start High Fidelity PCR system (Roche, UK). The PCR programme was initial denaturation at 95 °C for 2 min, then 30 cycles of denaturation at 95 °C for 30 s, annealing at 55 °C for 30 s and extension at 72 °C for 45 s, terminated by a final extension at 72 °C for 7 min. The DNA amplicons were then cleaned by AMPure bead purification reagent (Beckmancoulter, UK) to remove the residue of primers, enzymes and nucleotides. The cleaned DNA amplicons were individually quantified by dsDNA HS Assay Kit (Thermofisher, USA) on a fluorometer (Qubit 2.0, Thermofisher, USA). All the amplicons were diluted to equimolar 10 pM for building one pooled library. A template was prepared (includes amplification and enrichment) on Ion OneTouch™ 2 System (Life Technologies, UK). The templated Ion Sphere™ Particles obtained from template preparation was combined with an Ion 316™ Chip Kit v2 with 2 to 3millon reads per run for sequencing. The sequencing was conducted by Ion PGM™ Sequencing 200 Kit v2 (Life Technologies, UK) according to the manufacturer’s protocol. The data was generated as FASTQ file from ion serve. The Ion Torrent sequencing raw data is deposited in the NCBI Sequence Read Archive database (https://www.ncbi.nlm.nih.gov/biosample/13071290). The accession number is SAMN13071290. QIIME denoiser was then used to filter the sequencing data at a minimum length of 200 bp and to select the targeted gene according to the barcode. The operational taxonomic unit (OTU) table of anodic microbial communities was produced with the QIIME pipeline at 97% similarity level using the Greengenes database. All the sequence data were then rarified to a minimum sequencing read of 13,880 for the beta diversity analysis. Principal component analysis (PCA) was conducted on the OTU table at the family level and the post hoc Tukey-Kramer test (at 95% confidence level) was performed with STAMP (Parks et al. 2014).

To quantify the abundance of *Geobacteraceae*, quantitative PCR (qPCR) with forward primer *Geobacteraceae* 494f: 5′-*AGGAAGCACCGGCTAACTCC*-3′ and reverse primer *Geobacteraceae* 825r: 5′-*TACCCGCRACACCTAGT*-3′ were used (Holmes et al. 2002). All the extracted DNA samples were diluted with nuclease-free water 1:10, to minimize inhibition. Three microliters of diluted samples (1 × 10^8^ copies/μL to 1 × 10^2^ copies/μL) was individually reacted with a mixture of 5 μL of Ssofast EvaGreen Supermix (Biorad, UK), 0.5-μL forward primer, 0.5-μL reverse primer and 1-μLnuclease-free water and then was denaturized at 98 °C for 3 min, followed with 39 cycles for denaturation at 98 °C for 5 s, annealing at 51 °C for 10 s on a real-time PCR system (CFX96, Biorad, UK). To estimate the final cell numbers of *Geobacteraceae* in each sample, the copy number was assumed to be 2 per cell of *Geobacteraceae* (Methé et al. 2003).

### Data analysis and calculations

The electron equivalent of molecules (e^−^mmol/L) was based on:

2 $$ {S}_{e=}\ {S}_m\times N $$

*S*_*e*_ is the electron equivalent of molecules, *S*_*m*_ is the concentration of the molecules and *N* is the number of electrons released per molecule when the molecule is mineralised to CO_2_, protons and electrons. The number of electrons released per molecule when the molecule is mineralised to CO_2_, protons and electrons was shown in Table S2.

To calculate the rate of glucose degradation pathways in the glucose-acclimatized BESs, it was assumed that the reactions in the glucose-acclimatized BESs were in the first order, which subjected to the relation of that *r* = *k*[*R*], where *r* is the rate of reaction, *R* is the concentration of reactant, *t* was the reaction time and *k* is the rate constant. *k* was obtained based on the equation (Velasquez-Orta et al. 2011):

3 $$ lnR=- kt+{lnR}_0 $$

Linear regression and nonlinear regression were carried out by using Minitab 17 (Minitab Inc., USA).

## Results

### The effect of the acclimatization anode potential on glucose-acclimatized BESs

**Fig. 1:**
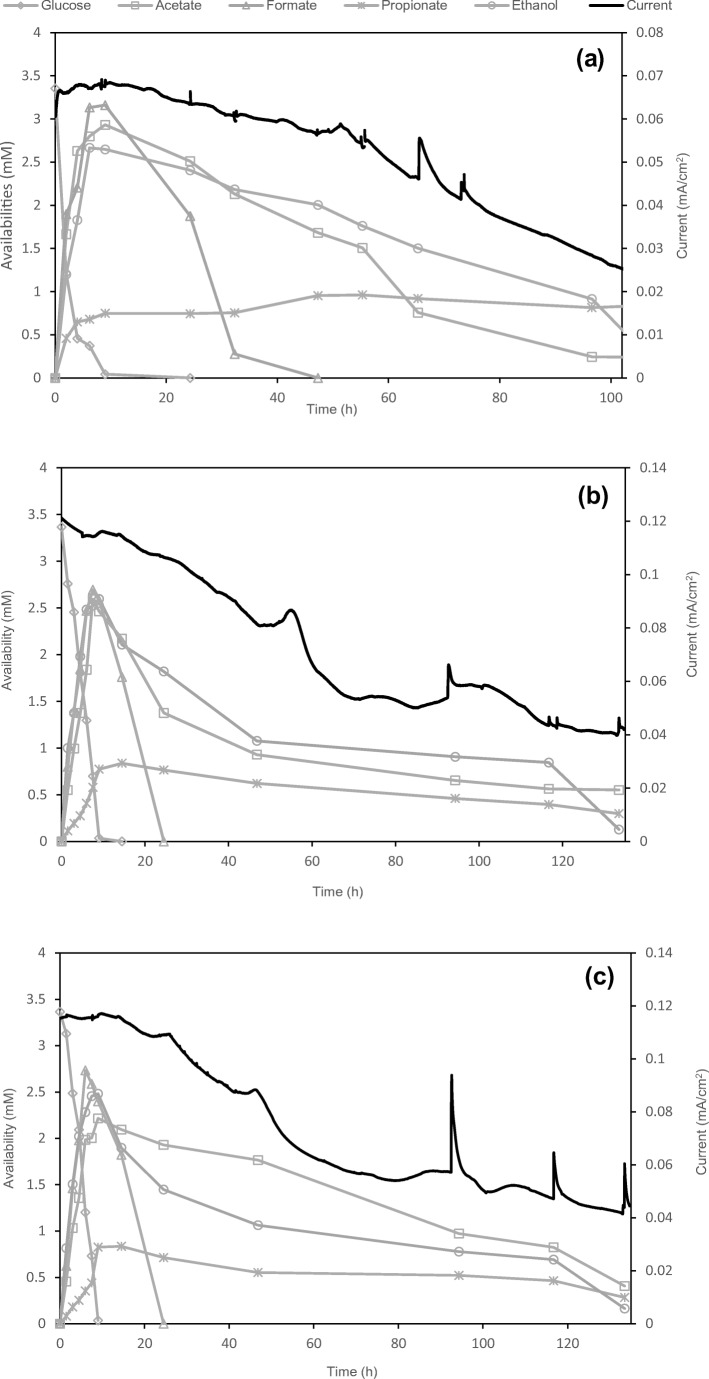
The degradation profile of glucose in the BESs acclimatized at **a** − 150 mV, **b** 0 mV, **c** + 200 mV

Glucose-acclimatized BESs at − 150 mV, 0 mV and + 200 mV were established to monitor the degradation process of glucose and current production at different anode potentials. This was done 2 weeks after the initial inoculation, after about five feeds, when the peak current was reproducible and stable. The initial acclimation anode potentials for each reactor were maintained during the whole experimental process. In a typical batch with 3.33 mM glucose, the current production peaked to ~ 0.12 mA/cm^2^ at the beginning of the tests in the 0-mV- and the + 200-mV-acclimatized reactors. When glucose was depleted after about 9 h, the current significantly decreased until < 0.05 mA in 124 h (Fig. 1). In the – 150-mV-acclimatized BESs, the peak current was only 0.07 mA/cm^2^ at the beginning of the test and decreased to < 0.03 mA/cm^2^ in 93 h (Fig. 1).

The degradation profile of glucose in these reactors was similar to the current: glucose degraded quickly in the first 9 h in all reactors. Formate, acetate, propionate and ethanol appeared as major intermediates and products during glucose degradation; they reached peak concentrations at around the 9th hour (Fig. 1). Subsequent consumption of formate was also rapid: formate was depleted by the 24th hour in the 0-mV- and +200-mV-acclimatized BESs, and by the 30th hour in the − 150-mV-acclimatized BESs. Acetate and ethanol were consumed at a slower rate and depleted by the end of the test when the current was very low (< 0.05 mA/cm^2^). The propionate concentration remained stable from the 9th hour onwards in the – 150-mV-acclimatized BESs, while in both the 0-mV- and + 200-mV-acclimatized BESs, it decreases slightly after this time(Fig. 1). Neither H_2_ nor CH_4_ was detected during the first 9 h, but both were detected in low amounts in all the reactors at the end of the runs, accounting for < 1% of the total electron equivalent of the initial glucose feed.

### Identification of the major electrogenic pathways and electrogenic barriers in glucose-acclimatized BESs

**Fig. 2:**
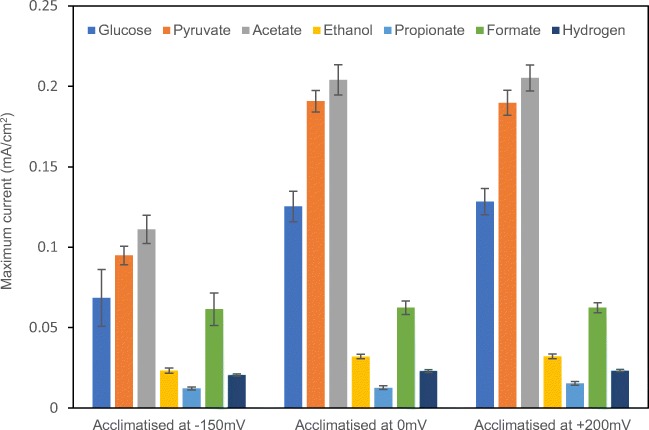
The current production from glucose and from its degradation products in glucose-acclimatized BESs acclimatized at anode potentials of − 150 mV, 0 mV and + 200 mV at testing anode potentials of − 150 mV, 0 mV and + 200 mV, respectively (se, *n* = 2)

To identify the major electrogenic compounds and their production and consumption routes, BES reactors were fed individually with acetate, formate, ethanol, propionate, pyruvate and hydrogen in both open and closed circuit mode. Major current production was observed with pyruvate, acetate and formate, while current production with ethanol, propionate and hydrogen was relatively low (Fig. 2). In the pyruvate-fed tests, pyruvate was broken down into acetate and formate (Table 1). High current continued to be produced when pyruvate was almost depleted (Fig. S1). This indicates that the current was produced by acetate and formate, while pyruvate itself was not a major direct substrate for current production. Coulombic efficiency is not directly related to current production, but an informative indicator of the efficiency with which the substrates give rise to current. The coulombic efficiency of these tests is reported in Table S1.

**Table 1:** The end products in 9-h individual tests of glucose degradation intermediates and products in glucose-acclimatized BESs

	The availability in closed circuit (mM)					The availability in open circuit (mM)				
	Acetate test	Ethanol test	Propionate test	Formate test	Pyruvate test	Acetate test	Ethanol test	Propionate test	Formate test	Pyruvate test
− 150 mV										
Acetate	–	0.15	< 0.01	n/a	4.20	–	0.10	< 0.01	n/a	3.57
Ethanol	n/a	–	n/a	n/a	n/a	n/a	–	n/a	n/a	n/a
Propionate	0.02	n/a	–	n/a	0.49	n/a	n/a	–	n/a	0.43
Formate	n/a	0.12	< 0.01	–	2.09	n/a	0.02	< 0.01	–	2.31
Hydrogen	n/a	n/a	n/a	n/a	n/a	n/a	n/a	n/a	n/a	n/a
0 mV										
Acetate	–	0.14	< 0.01	n/a	4.19	–	0.11	< 0.01	n/a	3.27
Ethanol	n/a	–	n/a	n/a	0.41	n/a	–	n/a	n/a	n/a
Propionate	0.01	n/a	–	n/a	0.47	n/a	n/a	–	n/a	0.33
Formate	n/a	0.08	< 0.01	–	2.11	n/a	0.02	< 0.01	–	2.26
Hydrogen	n/a	n/a	n/a	n/a	n/a	n/a	n/a	n/a	n/a	n/a
+ 200 mV										
Acetate	–	0.15	< 0.01	n/a	4.16	–	0.12	< 0.01	n/a	3.70
Ethanol	n/a	–	n/a	n/a	n/a	n/a	–	n/a	n/a	n/a
Propionate	0.01	n/a	–	n/a	0.55	n/a	n/a	–	n/a	0.45
Formate	n/a	0.12	< 0.01	–	2.00	n/a	0.02	< 0.01	–	2.49
Hydrogen	n/a	n/a	n/a	n/a	n/a	n/a	n/a	n/a	n/a	n/a

*n/a*, not applicable as production was less than 0.001 mM or not detected

To obtain the concentrations of acetate and formate at which maximum current production occurs, further tests were performed with these substrates. Current production increased with concentration until a saturated level was reached, adhering to the Monod equation–like curves (Figs. S2 and S3). Maximum acetate-based current production was reached at 6.2 mM, 6.4 mM and 8.6 mM of acetate, while formate-based current production reached a stable value at 6.9 mM, 8.9 mM and 8.0 mM in the – 150-mV-, 0-mV- and + 200-mV-acclimatized reactors, respectively. However, when fed glucose, none of the reactors accumulated enough acetate or formate to reach these high levels. Switching the reactors to other anode potentials (+ 200 mV, 0 mV, − 150 mV, − 250 mV and open circuit potential) in individual glucose-fed tests did not increase the availability of acetate and formate (Fig. S4). Coulombic efficiency with acetate was substantially higher than with formate as feed (Fig. S5).

**Fig. 3:**
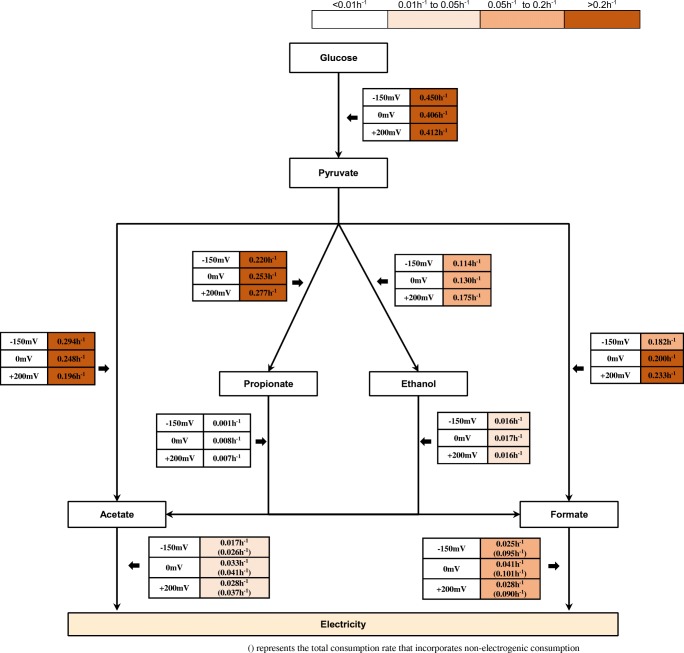
Glucose degradation and electrogenic pathways and conversion rates in glucose-acclimatized BESs at anode potentials of − 150 mV, 0 mV and + 200 mV. Details on the calculation of the conversion rates are given in the supplementary material S6

To find the rate-limiting steps in the conversion of glucose to acetate and formate and current, the concentrations of the various intermediates after 9 h of incubation were measured (Table 1). Irrespective of the anode, potential glucose was rapidly degraded via pyruvate (*k* = 0.407 h^−1^ to 0.450 h^−1^) to formate, acetate, propionate and ethanol (Fig. 3). The rate of acetate production from glucose (*k* = 0.196 h^−1^ to 0.294 h^−1^) was higher than the rate of acetate and formate conversion to current (*k* = 0.017 h^−1^ to 0.033 h^−1^ for acetate and *k* = 0.025 h^−1^ to 0.041 h^−1^ for formate) (Fig. 3). The rate of acetate or formate production from ethanol was equal to or lower than the rate of acetate and formate consumption for current production (*k* = ~ 0.017 h^−1^) (Fig. 3). The degradation of propionate was very slow in all the reactors (*k* = 0.001 h^−1^ to 0.007 h^−1^) (Fig. 3). Thus, the formation of the dead-end products of ethanol and propionate from glucose limited the generation of high current in these reactors.

### The relationship between the current production and anodic biofilm in glucose-acclimatized BESs colonized at − 150 mV, 0 mV or + 200 mV

**Fig. 4:**
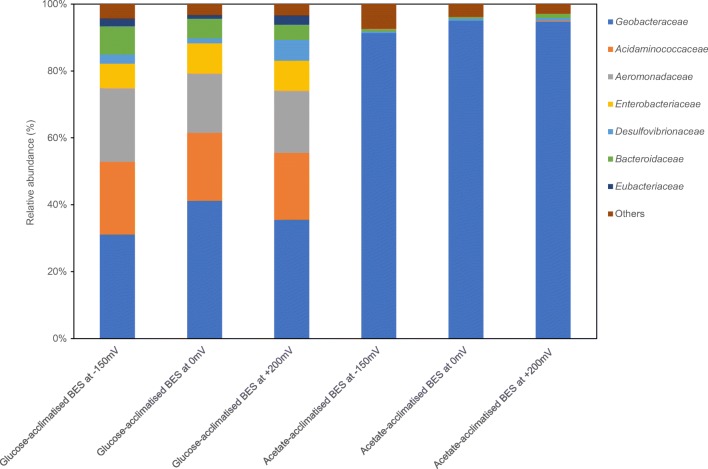
Summary of taxonomy of anodic microbial communities at family level relative abundance in glucose-acclimatized BESs and acetate-acclimatized controls

The relationship between the current production and abundance of the major electrogenic microorganism on the anode was analysed in detail, with special attention to *Geobacteraceae*. Acetate-acclimatized controls were used as benchmarks. Based on sequence recovery in acetate-acclimatized controls, *Geobacteraceae* comprised 87 to 92% of the biomass at the anode, which indicates a major electrogenic role of the members of this family. In glucose-acclimatized BESs, *Geobacteraceae* accounted for only ~ 29% in the – 150-mV-acclimatized BES; this percentage was slightly higher in the + 200-mV- and the 0-mV-acclimatized BESs, at ~ 35% and ~ 39%, respectively (Fig. 4). *Acidaminococcaceae*, *Aeromonadaceae* and *Enterobacteriaceae* were the next largest groups of these anodic microbial communities at about ~ 19%, ~ 18% and ~ 8%, respectively (Fig. 4). Principal component analysis (PCA) analysis indicated that the acclimation potential had a limited effect on the anodic microbial communities in both glucose-acclimatized BESs and acetate-acclimatized controls (Fig. S6).

**Fig. 5:**
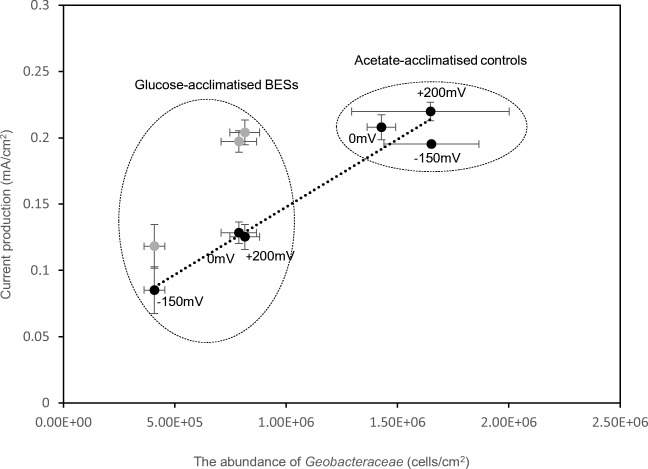
Current production with origin feed (black circle) or addition acetate (grey circle) versus abundance of *Geobacteraceae* in BESs (anode potential at 0 mV (Fig. S7))

The current production and the abundance of *Geobacteraceae* of the acetate-acclimatized controls were similar, irrespective of the acclimation potential, at ~ 0.21 mA/cm^2^ and ~ 1.5 × 10^6^ cells/cm^2^, respectively (Fig. 5). The abundance of *Geobacteraceae* in the glucose-acclimatized BESs was an order of magnitude lower, with only 4.0 × 10^5^ cells/cm^2^ in the 150-mV-glucose-acclimatized BES and ~8.0 × 10^5^ cells/cm^2^ in the + 200-mV- and 0-mV-glucose-acclimatized BESs (Fig. 5).

The current production of the reactors linearly increased with an increasing abundance of *Geobacteraceae* ranging from ~ 0.09 to ~ 0.21 mA/cm^2^ when the reactors were operated with their acclimation substrates (Linear regression, *p* < 0.05) (Fig. 5). This linear relationship broke down when 10 mM acetate was added on top of glucose in the 0-mV- and the + 200-mV-glucose-acclimatized BESs. The current production increased markedly and was higher than what would be expected based on *Geobacter* numbers alone; in fact, it was as high as in the acetate controls operating at the same potential (Fig. 5). Supplying additional acetate in the – 150-mV-glucose-acclimatized BESs also increased the current production, but only to ~ 50% of the levels in the corresponding acetate controls (Fig. 5).

## Discussion

Wastewater treatment in bioelectrochemical systems hinges on current production from a mixture of compounds, most of which are converted to intermediates before they can be used by electrogenic microorganisms. The efficiency of the electrogenic organisms producing current relies materially on the efficiency of the upstream stages of digestion. Our experiments show that even with a relatively simple substrate, glucose, these upstream pathways can be complex and sometimes unproductive; not all electrons are necessarily channelled into substrates that are converted to current.

At varied anode potentials, glucose was rapidly converted, mostly into acetate and formate that could be used by electrogenic microorganisms. However, regardless of anode potential, the rates of conversion of the upstream stages were suboptimal and not high enough to produce the current achievable when pure electrogenic substrates were used. An amount of glucose was always funnelled into ethanol and propionate; the rates of degradation of these were slow, further limiting the conversion of glucose into the current. This was especially clear towards the end of each batch when all glucose, acetate and formate had been depleted and only the remaining ethanol was yielding some current. A continuous glucose supply would be needed to provide constant current production and high coulombic efficiency, masking the slow rates of ethanol and propionate consumption. A continuous mode may be more suitable than batch-fed mode for BESs treating complex waste.

Current production in glucose-acclimatized BESs may also be increased by minimising the production of non-electrogenic molecules. It is therefore noteworthy that the pyruvate-fed runs did not produce much ethanol, producing higher peak availability of acetate and consequently current. Given that glycolysis results in the generation of pyruvate plus reducing equivalents (as NADH), which—unless removed—can give rise to the formation of non-electrogenic compounds such as ethanol; this suggests that removal of reducing equivalents generated during glucose degradation was limiting the rate and efficiency of current production (Lee and Rittmann 2009; Thauer et al. 1977). Removal of reducing equivalents can in theory be stimulated by tuning the anode potential, which makes it tempting to speculate that in continuously fed systems coulombic efficiency can be improved by tuning the anode potential. In our batch fed system, manipulating anode potential could not overcome the ethanol issue, probably because batch feeding overrode its effects.

Anode potential had little effect on the community composition of glucose-acclimatized BESs at the family level, where, irrespective of the acclimation potential, *Aeromonadacea* and *Acidaminococcaceae* were the major glucose fermenters (Chung and Okabe 2009; Hamberger et al. 2008; Speers and Reguera 2012). Zhu et al. (2014) also reported that the anodic microbial communities in their acetate-fed BESs were not significantly distinguished to the genus level by anode potentials. However, in various other studies, anode potentials did strongly select and shape the anodic microbial communities of BESs (Dennis et al. 2016; Ishii et al. 2014; Torres et al. 2009). Flow mode might be of significance here as both Zhu et al. (2014) and our study used batch-fed mode while others used continuous flow-mode.

Irrespective of the acclimation potential, the electrogenic microorganisms in our reactors were mainly *Geobacteracea* (Speers and Reguera 2012). *Geobacteraceae* numbers were linearly related to the current production in these reactors. This correlation may be spurious though: when supplied with additional acetate beyond that what was generated from glucose degradation alone, the glucose acclimatized reactors showed higher current production than what would be expected based on *Geobacteraceae* numbers alone. This suggests that in the glucose-acclimatized reactors, “non-*Geobacteraceae*” were also involved in current production. *Enterobacteriaceae* and *Aeromonadaceae* were observed in our reactors; these have been shown to produce low levels of current from acetate and glucose, respectively (Chung and Okabe 2009; Feng et al. 2014; Logan et al. 2019). It is likely that there were other presently unidentified electrogenic microorganisms active, as the gap between current production and what *Geobacters* can produce was in some cases substantial and too large to be explained by the presence and weak activity of *Enterobacteriaceae* and *Aeromonadaceae* alone. Another possible reason for the potentially spurious correlation is that the high ohmic and internal resistance of our H-type reactors was rate-limiting at higher current production levels; this resistance becomes more important as the current increases (Watson and Logan 2010).

Little is known about the extent to which the complex composition of wastewater impedes the implementation of new wastewater treatment technology such as BESs. The present study with a relatively simple complex substrate suggests that the effects of this complexity are huge and that current production from waste cannot simply be engineered via electrode potential alone. Unravelling the electrogenic pathway(s) of the complex substances present in wastewater, such as hydrolysable organics and suspended particles and their relationship to current production, energy production and coulombic efficiency are a challenge, yet urgently needed if BESs are to be implemented as cost-effective wastewater treatment systems.

## Electronic supplementary material

ESM 1 (PDF 779 kb)
