# Supplementary material for: The effect of anode potential on current production from complex substrates in bioelectrochemical systems: a case study with glucose
Source: Appl Microbiol Biotechnol. 2020 Apr 4;104(11):5133–43. doi: 10.1007/s00253-020-10547-6 (PMC7228986; doi:10.1007/s00253-020-10547-6)
Supplement: Supplementary file 1 — (PDF 779 kb) [file 253_2020_10547_MOESM1_ESM.pdf]

Applied Microbiology and Biotechnology

Supplementary Material

Title: The effect of anode potential on current production from complex substrates in bioelectrochemical systems: a case study with glucose

Fei Zhao, Elizabeth S.Heidrich, Thomas P. Curtis, Jan Dolfing

School of Engineering, Newcastle University, Newcastle-upon-Tyne, NE1 7RU England, UK

Corresponding Author:

Jan Dolfing

Address: School of Engineering, Newcastle University, Newcastle NE1 7RU, England (UK)

E-mail: jan.dolfing@ncl.ac.uk

Contents

S1 The example of a typical pyruvate-fed test in the glucose-acclimatized bioelectrochemical systems (BESs) 1

S2 The coulombic efficiency in the individual-fed tests in the glucose-acclimatized BESs in nine hours 2

S3 The evaluation of K_s of acetate and formate in the glucose-acclimatized BESs 3

S4 The effect of anode potential on the availability of glucose degradation intermediates and products when glucose was depleted after acclimation 4

S5 The optimal feed in the glucose-acclimatized BESs for electrogenic performance 6

S6 Calculation of the rate of glucose degradation pathway in glucose-acclimatized BESs 8

S7 The principle component analysis (PCA) of the anodic microbial communities 13

S8 A example of polarization curves of glucose-acclimatized BESs and acetate-acclimatized controls 14

S9 The design of the reactor and calculation of electron equivalent 15

S1 The example of a typical pyruvate-fed test in the glucose-acclimatized bioelectrochemical systems (BESs)

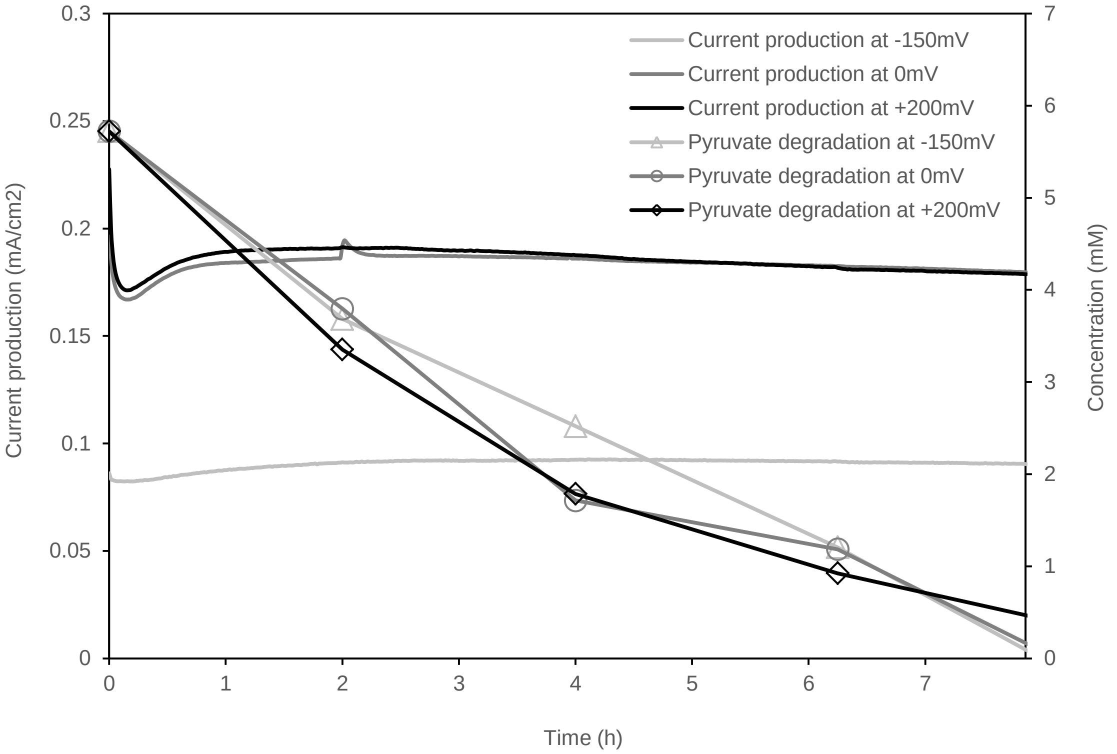

Fig. S1 A example of current production and pyruvate degradation in the pyruvate-fed tests in glucose-acclimatized BESs

S2 The coulombic efficiency in the individual-fed tests in the glucose-acclimatized BESs in nine hours

Table S1 The coulombic efficiency of the individual-fed tests in the glucose-acclimatized BESs in nine hours (%)

	+200mV acclimatized BESs	0mV acclimatized BESs	-150mV acclimatized BESs
Glucose cycle	22.3±3.3	18.4±2.8	11.0±2.0
Pyruvate cycle	46.9±2.5	45.8±3.1	30.4±3.0
Acetate cycle	73.3±3.0	80.5±1.2	75.2±6.4
Formate cycle	30.1±1.4	41.0±2.1	26.9±2.1
Ethanol cycle	65.6±16.2	37.5±20.9	55.9±21.5
Propionate cycle	N/A	N/A	N/A

N/A: the coulombic efficiency was not measured because the consumption of the total electrons was negligible

S3 The evaluation of K_s of acetate and formate in the glucose-acclimatized BESs

The value of *Ks* was evaluated based on the nonlinear regression with the function of Monod kinetics (equation 1) between the current and applied concentration (Fig. S2 and Fig. S3).

In -150mV-acclimatized reactor, K_s of acetate = 3.12mM, therefore saturated acetate for maximum current production = 3.12mM × 2 = 6.24mM.

In 0mV-acclimatized reactor, K_s of acetate = =4.23mM, therefore saturated acetate for maximum current production = 4.23mM × 2 = 6.38mM.

In +200mV-acclimatized reactor, K_s of acetate = 4.30mM, therefore saturated acetate for maximum current production = 4.30mM × 2 = 8.60mM.

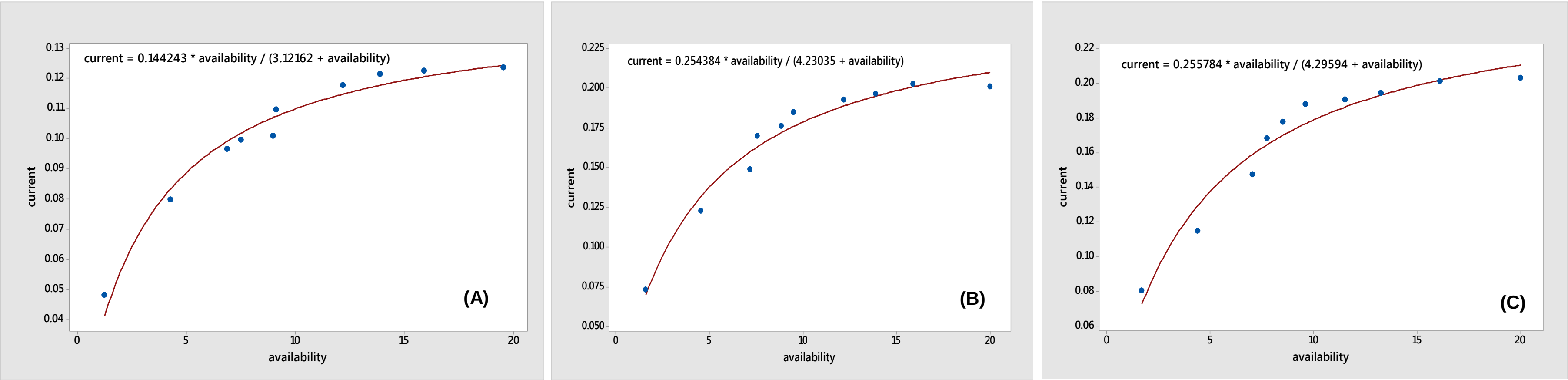

Fig. S2 The Monod-nonlinear regression curves of acetate base current production in glucose-acclimatized BESs at **A** -150mV. **B** 0mV. **C** +200mV

In -150mV-acclimatized reactor, K_s of formate = 3.44mM, therefore saturated acetate for maximum current production = 3.44mM × 2 = 6.88mM.

In 0mV-acclimatized reactor, K_s of formate = 4.46mM, therefore saturated acetate for maximum current production = 4.46mM × 2 = 8.92mM.

In +200mV-acclimatized reactor, K_s of formate = 3.99mM, therefore saturated acetate for maximum current production = 3.99mM × 2 = 7.98mM.

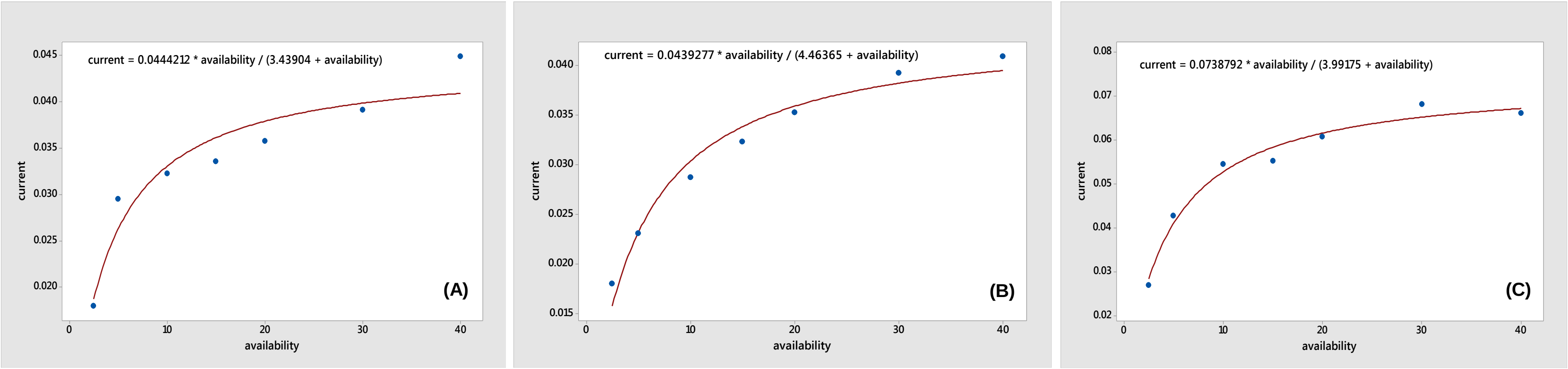

Fig. S3 The Monod-nonlinear regression curves of formate base current production in glucose-acclimatized BESs at **A** -150mV. **B** 0mV. **C** +200mV

S4 The effect of anode potential on the availability of glucose degradation intermediates and products when glucose was depleted after acclimation

All the reactors were exposed to five different testing anode potentials (+200mV, 0mV, -150mV, -250mV and open circuit) in five individual glucose-fed nine hour tests. Glucose was almost depleted after nine hours and acetate, formate, ethanol and propionate peaked around that time. The end products in each reactor at nine hour were shown in Figure S5.

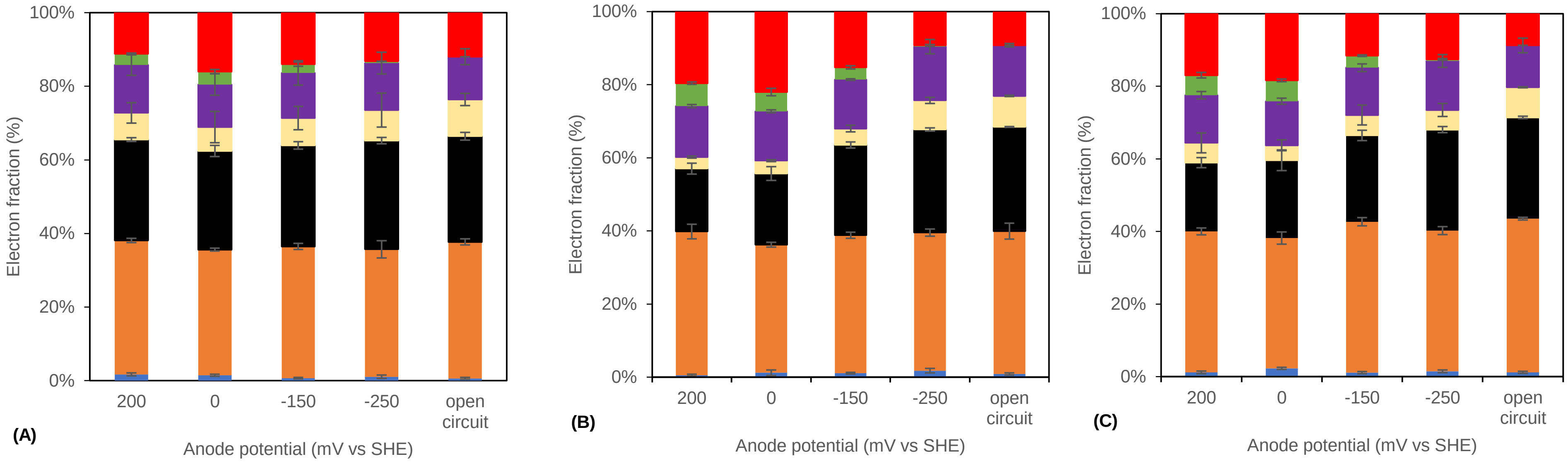

Fig. S4 The electron fraction of glucose (blue bar), ethanol (orange bar), acetate (black bar), formate (yellow bar), propionate (purple bar), current (green) and the consumption other than current production (red bar) in the effluent at the end of the nine hours glucose-fed tests with the testing anode potentials of +200mV, 0mV, -150mV, -250mV and open circuit respectively in the **A** -150mV-acclimatized BESs. **B** the 0mV-acclimatized BESs. **C** the +200mV-acclimatized BESs (se, n=2)

S5 The optimal feed in the glucose-acclimatized BESs for electrogenic performance

The optimal medium for the electrogenic performance of the glucose-acclimatized BESs was obtained here by feeding different mediums and were operated for nine hours. Acetate medium alone was optimal as with it the glucose-acclimatized BESs produced highest current and coulombic efficiency (Fig. S4).

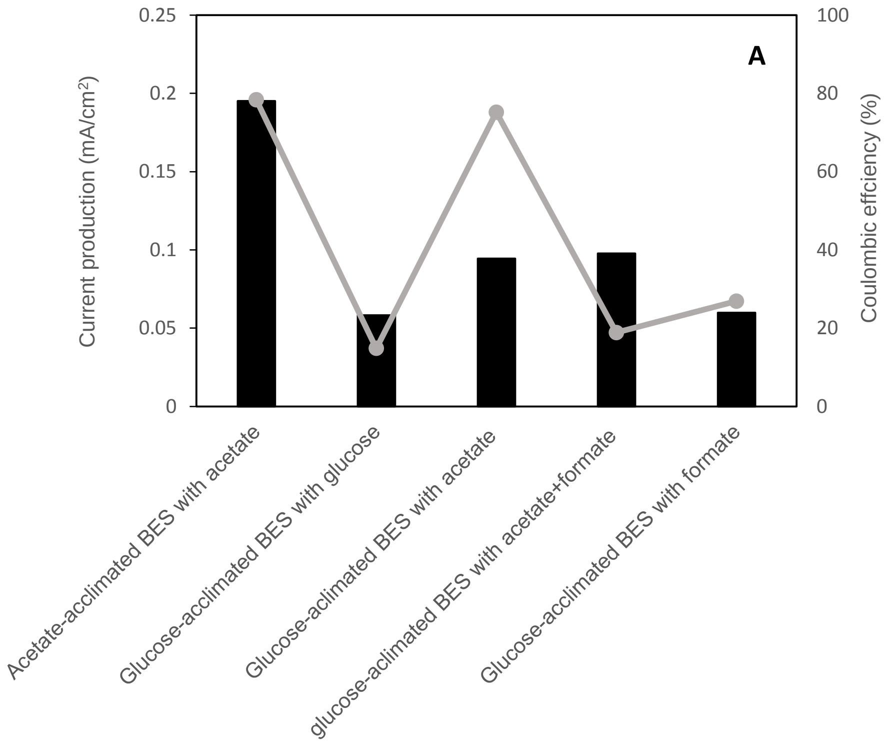

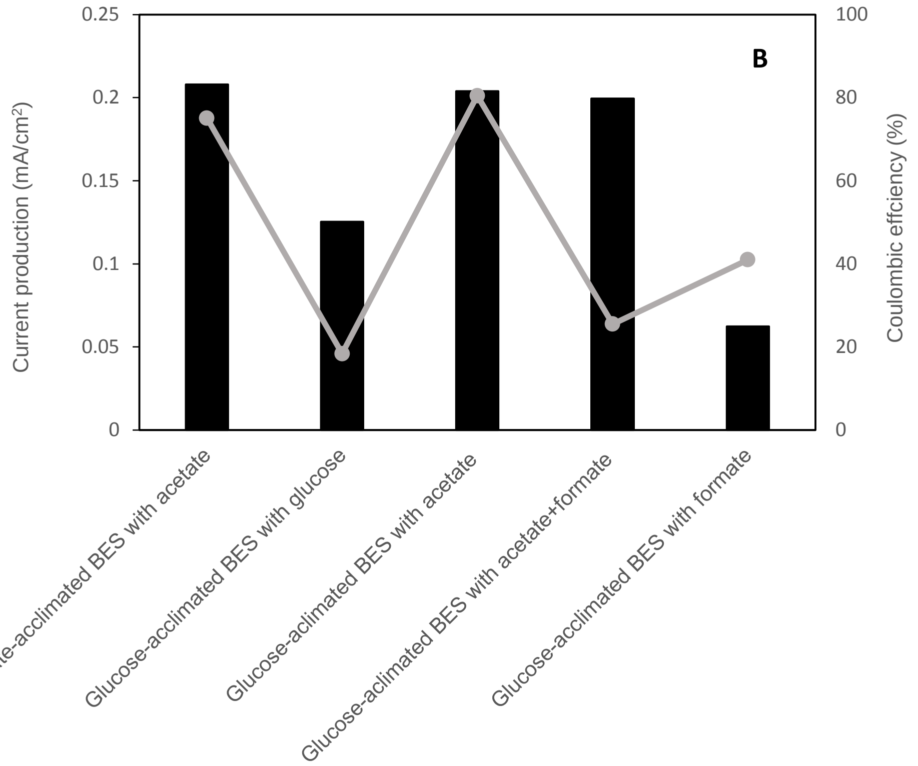

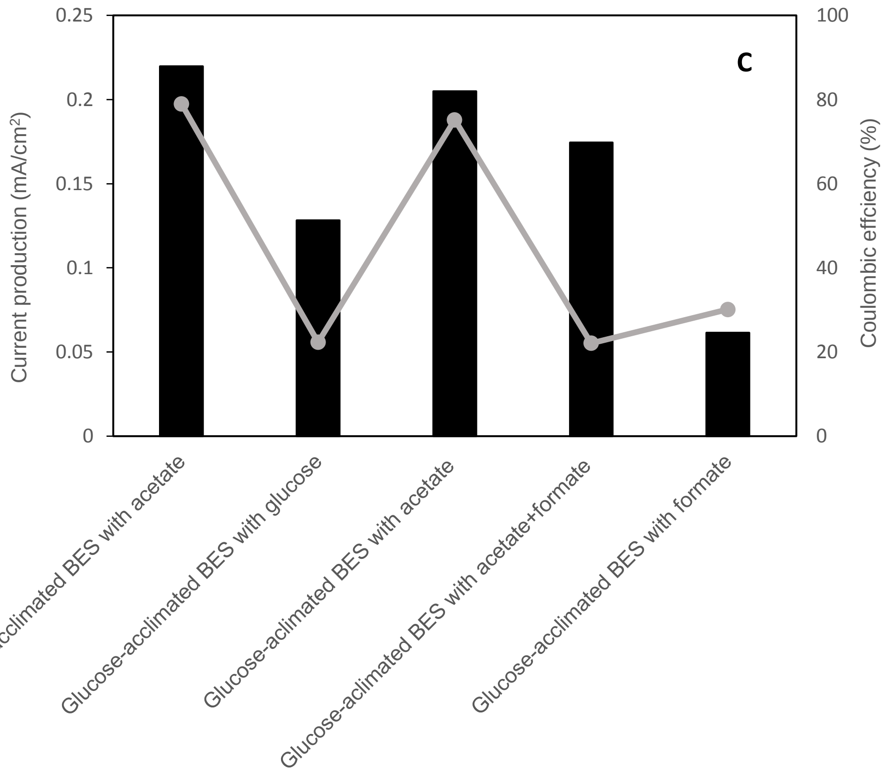

Fig. S5 The peak current production (black bar) and coulombic efficiency (grey line) of the glucose-acclimatized BESs with different mediums and the acetate-acclimatized controls acclimatized at **A** -150mV. **B** 0mV. **C** +200m

S6 Calculation of the rate of glucose degradation pathway in glucose-acclimatized BESs

The rate of glucose degradation pathway was estimated by linearly fit the logarithm of the reactant concentration and time based on equation 3. For glucose degradation rate:

-150mV

$$\ln(\text{glucose})=-0.450\text{time}+4.382$$

$$k=-0.450h^{-1}$$

0mV

$$\ln(\text{glucose})=-0.406\text{time}+5.045$$

$$k=-0.406\ h^{-1}$$

+200mV

$$\ln(\text{glucose})=-0.412\text{time}+5.109$$

$$k=-0.412h^{-1}$$

Ethanol and propionate were only produced from glucose degradation and the accumulation of ethanol peaked at 9 hours when glucose was depleted. Therefore, the decrease of ethanol or propionate from 9 hours until the end of the test approximately equal to the degradation rate of ethanol or propionate. In which case, the rate of ethanol or propionate degradation was calculated based on the regression of ln(ethanol) or ln(propionate) and time from 9 hours until the end of the test. In particular, for ethanol:

-150mV

$$\ln(\text{ethanol})= -0.0160\text{time}+3.789$$

$$k=-0.0160h^{-1}$$

0mV

$$\ln(\text{ethanol})=-0.0170\text{time}+3.557$$

$$k=-0.0170h^{-1}$$

+200mV

$$\ln(\text{ethanol})=-0.0158\text{time}+3.412$$

$$k=-0.0158h^{-1}$$

likewise, for propionate:

-150mV

$$\ln(\text{propionate})=0.001\text{time}+2.394$$

$$k=0.001h^{-1}$$

0mV

$$\ln(\text{propionate})=-0.008\text{time}+2.529$$

$$k=-0.008h^{-1}$$

+200mV

$$\ln(\text{propionate})=-0.007\text{time}+2.506$$

$$k=-0.007h^{-1}$$

Because acetate and formate was able to be produced from ethanol, the decrease of acetate and formate after 9 hour was not equal to the degradation rate of acetate and formate. In which case, the degradation rate of acetate and formate was obtained by the individual acetate-fed tests or individual formate-fed tests, where the degradation of acetate or formate was assumed as first order reaction when the concentration of them was low. In which case, the ln(acetate) and time was plot to evaluate the rate constant of acetate-based electrogenic processes.

-150mV

$$\ln(\text{acetate})=-0.026\text{time}+4.334$$

$$k=-0.026h^{-1}$$

0mV

$$\ln(\text{acetate}) = -0.041\text{time} + 4.420$$

$$k = -0.041h^{-1}$$

+200mV

$$\ln(\text{acetate}) = -0.037\text{time} + 4.554$$

$$k = -0.037h^{-1}$$

likewise, for formate,

-150mV

$$\ln(\text{formate}) = -0.095\text{time} + 2.244$$

$$k = -0.095h^{-1}$$

0mV

$$\ln(\text{formate}) = -0.101\text{time} + 2.548$$

$$k = -0.101h^{-1}$$

+200mV

$$\ln(\text{formate}) = -0.090\text{time} + 2.373$$

$$k = -0.090h^{-1}$$

Because the coulombic efficiency of acetate-based electrogenic processes in glucose-acclimatized BESs were 75.2%, 80.5% and 73.3% respectively in the glucose-fed BES acclimatized at -150mV, 0mV and +200mV (Table S1). Therefore, the rate of acetate-based electrogenic processes was :

-150mV

$$k = -0.023h^{-1} \times 73.3\% = 0.017$$

0mV

$$k = -0.041h^{-1} \times 80.5\% = 0.033$$

+200mV

$$k = -0.037h^{-1} \times 75.2\% = 0.028$$

likewise, the coulombic efficiency of formate-based electrogenic processes in glucose-acclimatized BESs were 26.2%, 41.0% and 30.1% respectively in the glucose-fed BES

acclimatized at -150mV, 0mV and +200mV (Table S1). Therefore, the rate of formate-based electrogenic processes was :

-150mV

$$k=-0.095h^{-1}\times 26.2\% = 0.025$$

0mV

$$k=-0.101h^{-1}\times 41.0\% = 0.041$$

+200mV

$$k=-0.090h^{-1}\times 31.1\% = 0.028$$

It was assumed that the total production of acetate, formate, ethanol and propionate from glucose degradation was proportional to the accumulation of these molecules.

In which case, for the rate of acetate production from glucose:

-150mV

$$\ln(acetate)=0.294time+1.132$$

$$k=0.294h^{-1}$$

0mV

$$\ln(acetate)=0.248time+1.219$$

$$k=0.248h^{-1}$$

+200mV

$$\ln(acetate)=0.196time+1.338$$

$$k=0.196h^{-1}$$

For the conversion rate of glucose to ethanol:

-150mV

$$\ln(ethanol)=0.114time+2.563$$

$$k=0.114h^{-1}$$

0mV

$$\ln(ethanol)=0.130time+2.435$$

$$k=0.130h^{-1}$$

+200mV

$$\ln(ethanol)=0.175time+2.225$$

$$k=0.175h^{-1}$$

For the conversion rate of glucose to formate:

-150mV

$$\ln(formate)=0.182time+0.527$$

$$k=0.182h^{-1}$$

0mV

$$\ln(formate)=0.200time+0.317$$

$$k=0.200h^{-1}$$

+200mV

$$\ln(formate)=0.233time+0.159$$

$$k=0.233h^{-1}$$

For the conversion rate of glucose to propionate:

-150mV

$$\ln(propionate)=0.220time+0.801$$

$$k=0.220h^{-1}$$

0mV

$$\ln(propionate)=0.253time+0.178$$

$$k=0.253h^{-1}$$

+200mV

$$\ln(propionate)=0.277time-0.089$$

$$k=0.277h^{-1}$$

S7 The principle component analysis (PCA) of the anodic microbial communities

Fig. S6 The principle component analysis (PCA) of the anodic microbial communities of the glucose-acclimatized reactors and the acetate-acclimatized controls acclimatized at -150mV, 0mV or +200mV

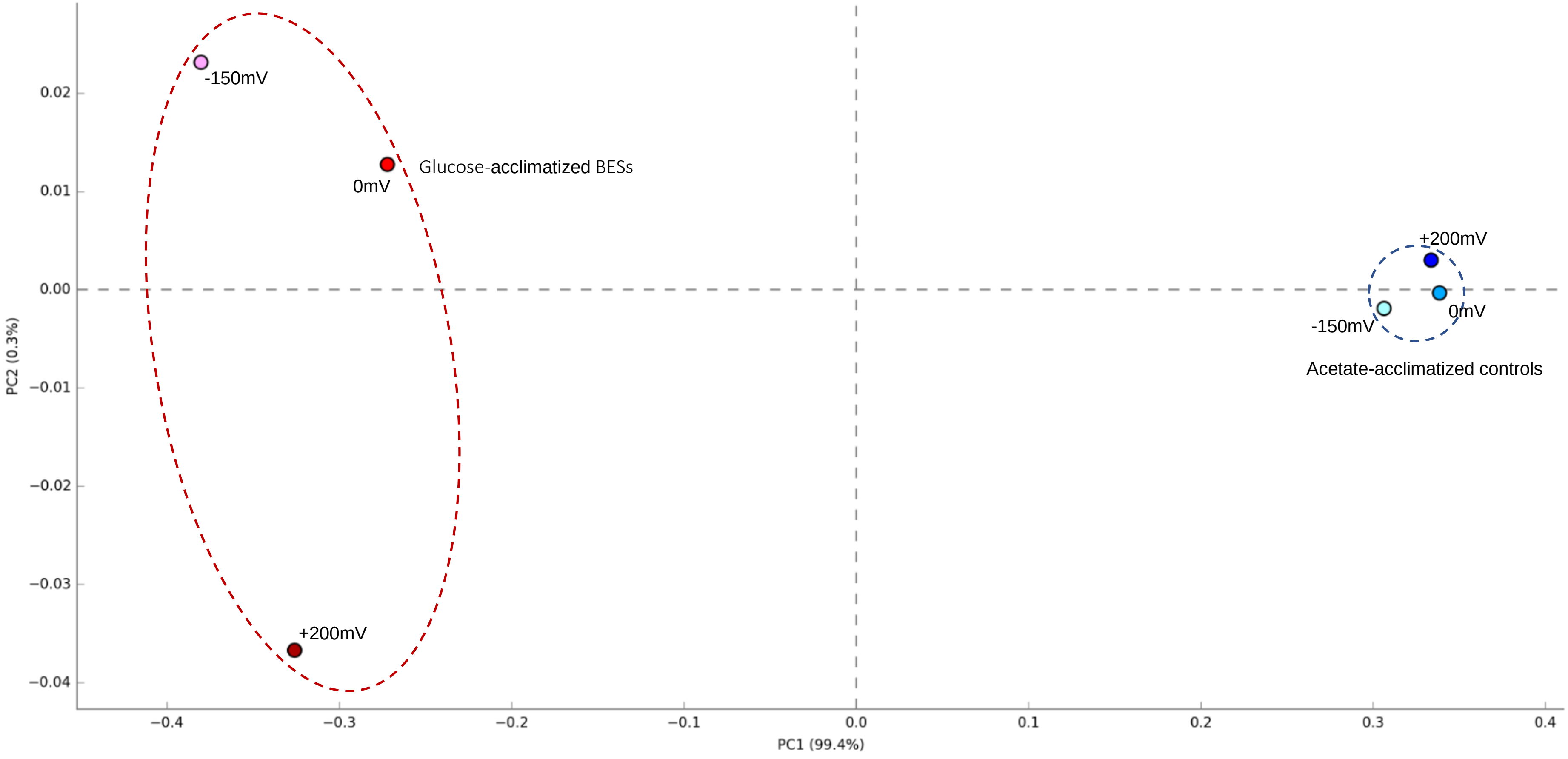

S8 A example of polarization curves of glucose-acclimatized BESs and acetate-acclimatized controls

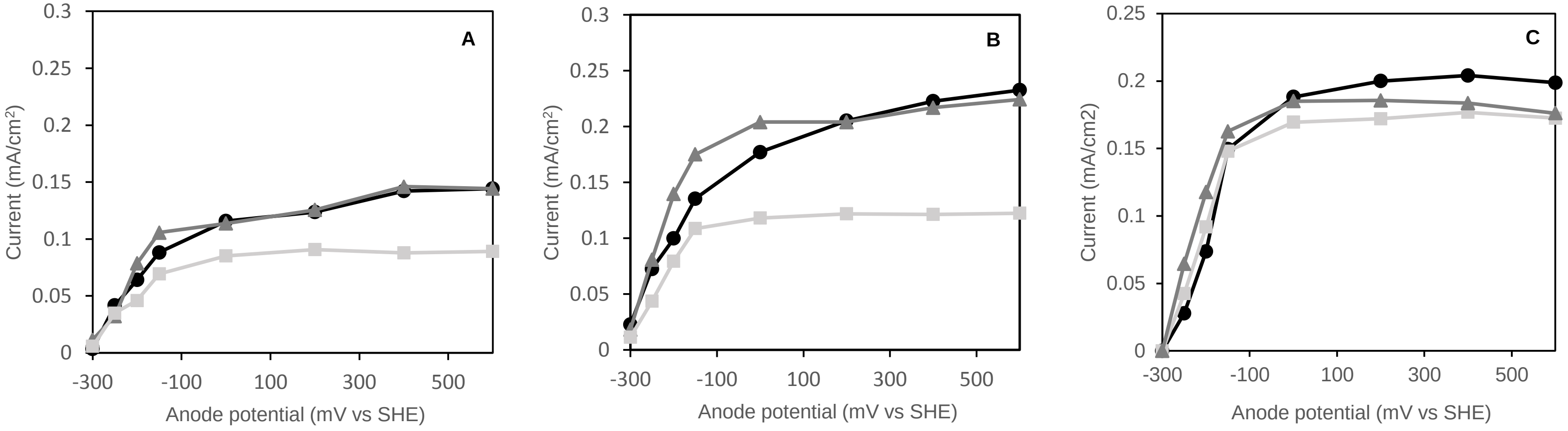

Fig. S7 Polarization curves of the glucose-acclimatized BESs at -150mV (light grey square), 0mV (dark grey triangle) and +200mV (black circle) in **A** 0.33mM glucose-fed cycles. **B** 10mM-acetate fed cycles. **C** the acetate-acclimatized controls with 10mM acetate

S9 The design of the reactor and calculation of electron equivalent

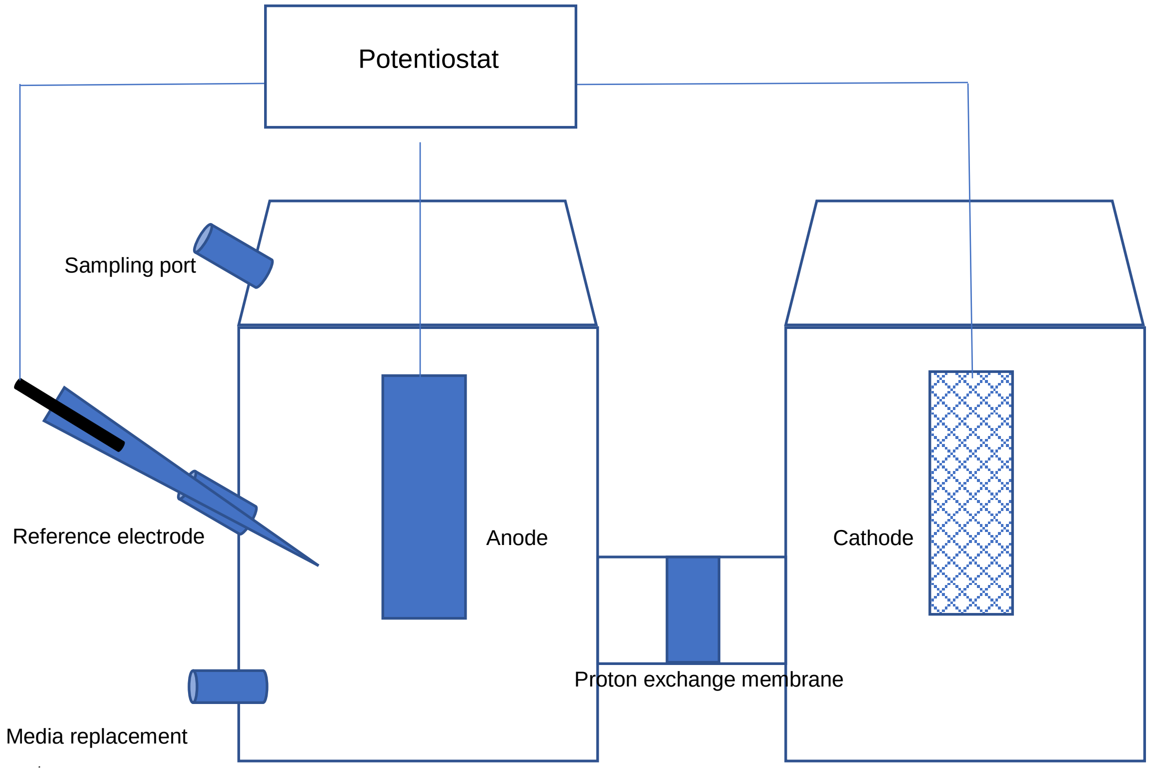

Fig. S8 The schematic description of H-type two-chambers BES reactor

Table S2 The electron equivalent of glucose and degradation intermediates and products

Molecules	Degradation reaction	Electron equivalent per carbon
Glucose	$C_6H_{12}O_6 + 6H_2O = 6CO_2 + 24H^+ + 24e^-$	24/6=4
Pyruvate	$C_3H_4O_3 + 3H_2O = 3CO_2 + 10H^+ + 10e^-$	10/3=3.33
Propionate	$C_3H_6O_2 + 4H_2O = 3CO_2 + 14H^+ + 14e^-$	14/3=4.67
Ethanol	$C_2H_6O + 3H_2O = 2CO_2 + 12H^+ + 12e^-$	12/2=6
Acetate	$C_2H_4O_2 + 2H_2O = 2CO_2 + 8H^+ + 8e^-$	8/2=4
Formate	$CH_2O_2 = CO_2 + 2H^+ + 2e^-$	2/1=2
